# Knowledge, help-seeking and efficacy to find respite services: an exploratory study in help-seeking carers of people with dementia in the context of aged care reforms

**DOI:** 10.1186/s12877-018-1009-7

**Published:** 2019-01-07

**Authors:** L. Phillipson, K. Johnson, E. Cridland, D. Hall, C. Neville, E. Fielding, H. Hasan

**Affiliations:** 1 School of Health and Society, Faculty of Social Sciences, University of Wollongong, Wollongong, Australia; 2 Australian Health Services Research Institute, University of Wollongong, Wollongong, Australia; 3 School of Nursing and Midwifery, Faculty of Health, Engineering and Sciences, University of Southern Queensland, Toowoomba, Australia; 4 Dementia Centre for Research Collaboration, Faculty of Health, Queensland University of Technology, Brisbane, Australia

**Keywords:** Carers, Dementia, Respite, Information seeking, Service use, System reform

## Abstract

**Background:**

Research highlights the need for carers of people with dementia to acquire relevant and timely information to assist them to access appropriate respite services. Unfortunately, negative experiences of information-seeking can create additional stress for carers and contribute to delays in up-take, or not using respite services at all.

**Methods:**

Cross-sectional survey data was collected from a convenience sample of *n* = 84 carers of older people with dementia living in the Illawarra-Shoalhaven region of NSW, Australia. We assessed knowledge, attitudes, information seeking behaviours, and unmet need for respite services in 2016, following national aged care reforms.

**Results:**

Over the previous 12 months, 86% of carers sought respite service information. The majority (73%) of all carers reported an unmet need for respite services, and were relying on personal networks to provide support for respite information. Few utilised the new government gateway ‘My Aged Care’ phone line (11%) or website (25%). However, 35% used a pre-existing helpline to access short term or emergency respite. We found a preference for interpersonal information sources, including local doctor (65%), professionally and volunteer led carer support groups (49%), and family and friends (46%). Those using four or more information sources showed higher capacity to name local respite services. Respite service information seekers were more likely to be caring for someone with behavioural problems, to have received assistance to access services, and to have used respite services in the past 3 to 6 months.

**Conclusions:**

New reforms in the Australian aged care sector have not adequately responded to the needs of carers of people with dementia for respite service information and support. Wider, community-based messaging promoting positive service options and the provision of active personal support is required to address the unmet need for respite in carers of people with dementia.

## Background

In 2015, an estimated 47 million people were living with dementia throughout the world, with this number expected to triple by 2050 [1]. In Australia, dementia affects almost 1 in 10 people aged 65 and over, with estimates of 354,000 in 2016, and its prevalence increasing by 40% from 2006 to 2016 [2]. Dementia is a degenerative neurological syndrome which impacts on multiple areas of function including memory, communication, and ability to perform activities of daily living [3]. While some people with dementia require institutional care, the vast majority live at home [3, 4]. Having a co-resident carer increases the likelihood that people with dementia will live at home longer [5]. Formal services in the community can assist people with dementia and their carers via: help with home duties; support through counselling services and support groups; and the provision of ‘respite’ services or substitute care [6]. Respite as an outcome can be achieved through the use of multiple strategies. In this paper, we are focused on engagement with formal respite services which typically include planned and emergency services in-home, centre-based, out-of-home or short stays in residential care services [7].

**Fig. 1:**
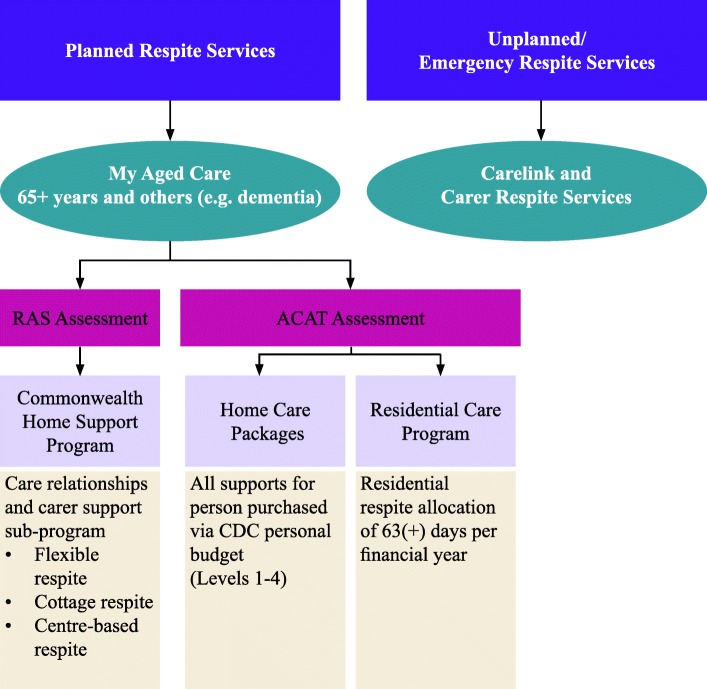
Respite in the Australian aged care system

In the Australian context, access to all service types is possible. However under new reforms, this occurs via a number of different pathways and programs (see Fig. 1). Respite service use provides carers with a break to attend to their own health or social needs, and can also enable people with dementia to participate in meaningful and appropriate activities or opportunities for social engagement and stimulation [8, 9]. Consumer directed care systems (where the focus for funding of supports is tied to meeting the specific goals of the person with the disability) enforce this emphasis with carer benefit only positioned as a ‘secondary’ or indirect outcome of service use by the person with the disability [10].

Despite respite being consistently identified by carers of people with dementia as one of their critical unmet care needs [11], the overall proportion who use respite and other support services is low [12, 13]. Research has highlighted a number of factors leading to low uptake of respite services including beliefs regarding poor service quality, guilt or the possibility of negative outcomes, a confusing and fragmented dementia services care environment, lack of availability and flexibility in service provision, and poor communication and referrals to support services [13, 14].

### Knowledge of respite services and efficacy to access

Carers of people with dementia are often responsible for proxy help-seeking and care related decision making on behalf of the person with dementia [15] as well as for personal help-seeking to find support for their own needs [14]. With regards to respite seeking, previous research highlights the critical need for carers to access appropriate information about the right services, at the right time [13, 16–19]. Knowledge of the service system has been associated with generic respite service use and non-use [18, 20], and non-use of centre-based respite [13].

Sourcing information about respite can add to the stresses associated with caring for someone with dementia. Robinson et al. [16] describe the mental stress and emotional turmoil for carers of people with dementia searching for service information in a complex system. Such negative experiences of information-seeking can lead to carers either delaying up-take, or not using respite at all [11, 16, 21].

Lack of awareness or confusion about the range of services available can inhibit early take-up of services, and, in turn, contributes to a sense that respite services should only be used as a last resort [12, 22]. The fact that ‘respite’ has also often been accessed through numerous funding programs, with differing eligibility and fee requirements, can be confusing for the carer and the person with dementia. This confusion can preclude early use and hinder information about available services or support in care planning, including how respite could be accessed in an emergency [12, 22].

In Australia, as part of the broader ‘Living Longer Living Better aged care reforms [23], a single gateway service for older people called ‘My Aged Care’ was introduced in July 2013 [24]. This program includes a telephone helpline and website to assist with information and referral but does *not* include the option for access to a single ‘named’ contact which has been identified as important for people living with complex needs such as those with dementia [17].

Australian reforms also supported changes to the administration of planned respite services, with an amalgamation of funding for planned respite services from the previous National Respite for Carers Program into the new Commonwealth Home Support Program [9, 25]. Subsequently, in December 2015, a program of reform for carer supports commenced, including the introduction of a single Carer Gateway (website and telephone line) to promote carer access to information, education, and counselling services [26]. At this stage, access to short term and emergency respite remains with the Commonwealth Carer Respite Centres (CCRCs) who previously provided carer information, education, counselling and arrangements for emergency respite services as well as referral for planned respite [27]. Unfortunately, for carers of people with dementia, the reforms now require them to negotiate with three different programs: My Aged Care for planned respite services [25], CCRCs for short term and emergency respite [27]; and the Carer Gateway for information, education and counselling [26]. While the reforms in Australia aim to improve services for carers and people with dementia, they may lead to increased confusion as people attempt to navigate a system in transition [9]. Currently, the impact of these reforms on provision of information and support to access respite for carers of people with dementia is unknown.

### Research objectives

This study aimed to explore the knowledge, information seeking behaviours, and perceived need for respite of carers of people with dementia following aged care reforms in Australia. Of particular interest was carers’ knowledge of local respite services; where carers sought information about local respite services; their rating of the usefulness of these sources; and, who (if anyone) helped them find information. The study also has international significance given the increased transition to consumer directed care systems in the UK, US, and parts of Europe [28].

## Method

### Survey

The survey tool utilised a number of standardised scales. Variables assessed included demographic characteristics (e.g. gender, age, education, languages others than English; caregiver relationship, financial status) as well as knowledge of respite services [29]. We also assessed carers’ perceptions of the level of the person’s cognitive disability [30]. Factors which impact on service use were assessed (e.g. receiving caregiver training). Finally, carers’ need for respite was assessed using both perceived and evaluated need standardised scales including: Role Captivity [30], Zarit Burden scale [31] and Family Caregiver Self-efficacy for Managing Dementia [32], as well as current and intended use of respite services, and where respondents looked for information. Problematic Behaviour for the person with dementia [30] was also used as an indirect measure of carer need for respite.

### Sample

**Fig. 2:**
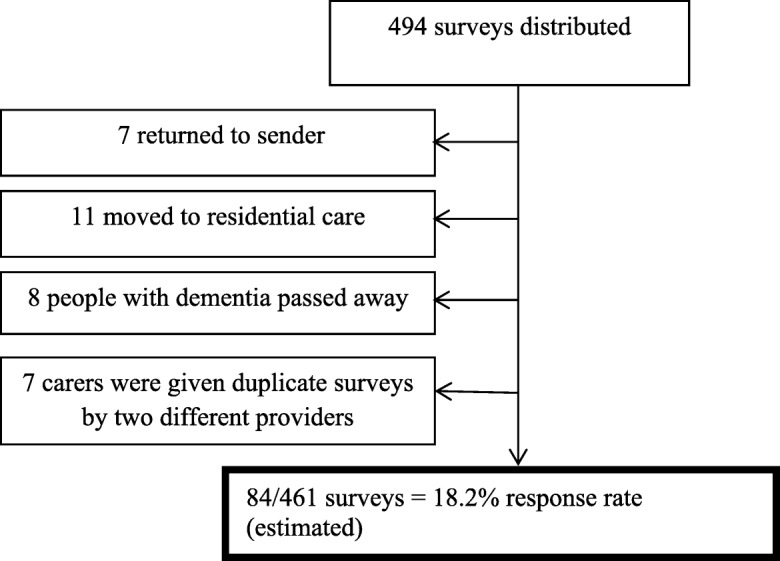
Survey responses

There is no accurate sample frame for carers of people with dementia in the community in Australia. As such, convenience sampling of help-seeking carers was conducted using community and service provider channels in the study region. These included: the CRCC, Aged Care Assessment Teams (ACAT), respite service providers, and carer support groups. Data collection was conducted from September 2015 to July 2016. A total of 494 paper surveys were distributed. Overall, eighty-four of a possible 461 surveys were returned, resulting in an estimated response rate of 18.2% (the rate may be higher due to distribution to the same carer by multiple services, or non-compliance with survey distribution by services). Thirty-three returned surveys were excluded as ineligible. Seven duplicates were identified in the returned sample and were excluded. See Fig. 2.

### Analysis

Simple descriptive analysis was utilised to describe the characteristics of those seeking information for respite. As some respondents had random missing data, data imputation was conducted in cases where less than 20% of data was missing within responses for a particular scale, specifically on the scales of Cognitive Status (8 participants) [30], Problematic Behaviour (13 participants) [30], and Family Caregiver Self-efficacy for Managing Dementia (2 participants) [32]. The mean score for a given respondent on a specific scale was manually imputed. Relationships between categorical variables were analysed through Chi-square test for independence or Fishers exact test (for tests with a 2 by 2 table or where the minimum expected cell frequency assumption was violated). To examine relationships between groups on continuous variables, t-tests were used for those variables with normal distributions and Mann-Whitney U tests for those with non-parametric data. Significance was set at *p* = .05 with Bonferroni adjustment for multiple comparisons.

## Results

### Demographic characteristics

The demographic characteristics of carers and of the person with dementia are provided in Table 1. The sample represented both male and female carers and people with dementia, as well as different types of carer relationships (e.g., spousal and non-spousal caregivers). The majority of care givers were female and lived in the same house as their family member or friend (person with dementia). Most of the people with dementia reported a medical diagnosis of dementia, with the majority having a primary diagnosis of Alzheimer’s disease. Many respondents reported an unmet need for respite. Many carers were receiving support from formal services and/or from family and friends, however 18% received no support from either source. Despite most receiving some form of support, many carers reported an unmet need for respite services. See Table 1.

**Table 1:** Characteristics of study participants

Demographic variable	Total *n* = 84
Carer	
Age^a^, Mean (range)	70 (38–92)
Gender^a^. Female *n* (%)	62 (74%)
Language spoken at home^a^, English *n* (%)	72 (86%)
Relationship, spousal *n* (%)	59 (70%)
Person with dementia	
Age^a^, Mean (range)	79 (61–94)
Gender^a^. Female *n* (%)	32 (39%)
Language spoken at home, English *n* (%)	68 (83%)
Medical diagnosis, yes (*n* (%)	74 (90%)
Type of dementia, *n* (%)	
Alzheimer’s	42 (56%)
Vascular	12 (16%)
Lewy Body	3 (4%)
Other	9 (12%)
Unknown	9 (12%)
Pearlin Cognitive Status^b^, ^a^Mean, SD	2.42 (0.64)
Respite knowledge and behaviours	
Tried to find respite information, yes *n* (%)	71 (85%)
Knowledge, Ability to name three respite services^a^, yes *n* (%)	42 (51%)
Receive some support (formal or informal), yes	67 (82%)
Mean (SD), number of respite services used in past 3–6 months	1.32 (0.93)
Would like more respite^a^, yes	51 (68%)

*Note.* ^a^Valid percent is reported due to missing data. ^b^8 items, range 0 (Not at all difficult) to 5 (Can’t do at all)(higher score denoting poorer perceived function)

### Respite information seeking behaviours of carers

The majority of respondents (*n* = 71, 86%) reported actively seeking respite information during the past 12 months. Of those who had looked for information about local respite services, the majority or many sought information from their general practitioner (GP) or local doctor, with slightly less than half using either volunteer or support carer groups or family or friends. Just over a third had utilised a government-funded helpline (CRCCs) which is the access point for emergency respite. Only 16% had utilised the My Aged Care helpline, however most rated it as an excellent or good information source. Other sources (27%) were reported as specific service providers, local councils, specialist doctors and ACAT. It should be noted that the three highest used sources of information were person to person sources, with the fourth highest a helpline rather than internet source. Only one quarter had utilised the My Aged Care website (the new portal for accessing aged care services including planned respite) with 72% rating it as an excellent or good source of respite information. Apart from My Aged Care, other internet sites were rarely utilised as information sources about respite. The majority of respondents rated information sources as good or excellent, with the exception of a Carers NSW helpline and website, and a CRCC website which has now been discontinued. See Table 2.

**Table 2:** Carers’ scoring of sources of respite information

Source (*n* = 68)	Type	Excellent/Good	Fair/Poor	Total
Your GP or local doctor	Person	34	12	46 (65%)
Carer support group	Person	33	2	35 (49%)
Family or friends	Person	26	7	33 (46%)
Commonwealth Respite and Carelink	Helpline	23	2	25 (35%)
My Aged Care	Website	13	5	18 (25%)
Other ‡	Person	16	3	19 (27%)
My Aged Care	Helpline	7	1	11 (16%)
Dementia Advisory Service	Person	7	1	11 (16%)
Alzheimer’s Australia National Dementia Helpline	Helpline	5		9 (13%)
Carers NSW	Helpline		3	5 (7%)
Dementia Illawarra Shoalhaven	Website	2		4 (6%)
Carers NSW	Website	1	2	4 (6%)
Commonwealth Respite and Carelink	Website	1	1	3 (4%)
HSNet	Website			0 (0%)

*Note.* ‡ ‘Other’ included specific service providers, local councils, specialist doctors and Aged care services

A number of carers reported looking for information from more than one source: 33 (47%) used two-three sources and 27 (38%) four or more sources. The remaining carers, 11 (16%) used only one source. Those using four or more sources were more likely to be able to name three respite services [78% (4 or more sources) vs. 46% (one source) or 42% (2 to 3 sources), χ^2^ = 8.172 (2), *p* = .017].

### Relationship between types of support and respite knowledge

Respondents were asked to name three respite services or facilities within their local area. Forty-two (52%) respondents named three respite services, 31 (38%) could name one to two services, and 10 (8%) could not name any respite services within their local area. It was hypothesised that those who were currently receiving support from formal or informal sources, would have a higher knowledge of respite services. Knowledge of local respite services was not related to having received carer training or being a member of a support group. Those who reported they had assistance to access services were significantly more likely to be able to name three respite services (*p* = .007, Fisher’s Exact Test). See Table 3. Of those *n* = 53 who had assistance, 85% reported formal sources (doctors, social workers, respite services), 9% reported informal sources (family or friends), and 6% reported both formal and informal sources.

**Table 3:** Relationship between types of support and knowledge of respite services

Carer Characteristics	Total, *n* = 81	Named 3 services *n* = 42	Named < 3 services *n* = 39	P*
Formal support, yes^a^	50 (64.9%)	30 (75.0%)	20 (54.1%)	.061
Informal support, yes^a^	40 (52.6%)	27 (65.9%)	13 (37.1%)	.021
Caregiver training, yes	43 (53.1%)	21 (50.0%)	22 (56.4%)	.658
Carer Support Group, yes^a^	40 (50.0%)	20 (52.6%)	20 (47.6%)	.823
Assist to find services (navigate), yes^a^	53 (77.9%)	33 (91.7%)	20 (62.5%)	.007

*Note.* Cell values represent frequencies (%) for categorical variables. All analysed using Fisher’s exact test. *Significance set at *p* ≤ 0.01 due to Bonferroni correction. ^a^Valid percent is reported due to missing data

### Relationship between ever seeking information and perceived or evaluated need

With regards to perceived or evaluated ‘need’, information-seekers reported significantly higher scores for their family member or friend’s problem behaviours (*U* = 161.00, Z = − 2.98, *p* = .003). See Table 4.

**Table 4:** Need factors associated with ever seeking information about respite

Need factors	Total *n* = 83	Yes *n* = 71	No *n* = 12	P*
Would you like more respite than you are currently receiving^a^, yes	50 (68%)	46 (72%)	4 (40%)	.068
CG Zarit Burden Score^a b^ 4 items, 5 pt. scale, 1(Never) to 5 (Nearly always)	2.90 (85%)	2.96 (87%)	2.48 (56%)	.075
CG Role Captivity^a^ 4 items, 4 point scale 1 (Not at All) to 4 (Very Much)	2.02 (0.69)	2.06 (.72)	1.78 (.43)	.263
CG, Self-efficacy^a^ 10 items, 10 pt. Visual Analogue Scale	6.67 (1.82)	6.69 (1.79)	7.02 (2.06)	.301
PWD, behavioural problems 13 items, 4 pt. scale, 1 (No days) to 4 (5 or more days)	1.90 (0.55)	1.97 (.56)	1.48 (.29)	.003

*Note. CG* Caregiver, *PWD* Person with dementia. Continuous variables with Mann-Whitney U test unless otherwise labelled. *Significance set at *p* ≤ .0125 due to Bonferroni correction. ^a^Valid percent is reported due to missing data. ^b^Analysed via t-test

## Discussion

The vast majority of carers reported they had tried to find information about local respite services in the past 12 months. Whilst most had some knowledge of local respite services, almost 10% could not name any respite services within their local area. Very few had utilised the new service gateway website or telephone helpline. Of those that had, a higher proportion rated the phone helpline as excellent or good, compared to the website. The three highest used sources of information were person to person sources, with the fourth highest a helpline rather than an internet source.

Significantly, carers of people with dementia benefitted not just from encouragement but also from active support to find respite service information. This may relate to the significant burden that navigating service pathways adds to their role as carers [33]. Previous research has highlighted that carers and people with dementia value and benefit from guidance to make decisions around respite [33–36]. While carers in this study reported using GPs, practice nurses and family and friends for information support, the fact that high knowledge was associated with having used three or four sources of information suggests the need to improve access to both comprehensive respite information, as well as formal (system) support. The carers in this cohort were mostly elderly themselves. As such, limitations in physical mobility as well as their significant caring responsibilities would likely have reduced opportunities to physically interact with interpersonal sources for information including their peers, their local doctor or members of their extended family.

Overall, almost three quarters of carers in the study expressed an unmet need for respite. This result highlights the importance of respite for carers of people with dementia and the desire for respite as a primary outcome for themselves, not only as a secondary outcome of service use by the person they care for. This was particularly so for those carers supporting people with behavioural issues. Unmet need for respite has been highlighted in previous Australian and international studies [13, 37]. However, this study suggests that the new reforms in the Australian aged care sector may be inadequate in prioritising the unique need for respite services by people with dementia.

### Implications for policy and practice

To better serve carers of people with dementia, this study highlights the need for promotional strategies using a wide variety of channels to provide respite service information. Greater promotion of new ‘gateway’ services, especially telephone helplines for respite information are also needed. Given the strong preference for interpersonal sources of information, the My Aged Care helpline could consider offering a named personal contact or ‘case worker’ whom carers of people with dementia can liaise with for follow up, rather than navigating their way through the Gateway system each time they call.

Offering a range of personal contact avenues (e.g. telephone, email, mail, and in-person) through multi-organisation directories may help to ensure simplified and accessible information about service pathways [38]. Also, given the importance of positive social norms for respite use, promotional messages should be aimed at the general public and also include the initial and continuous training of care providers. Specifically respite information dissemination should target informal support networks, the primary care sector and carer support groups who should see it as central to their role to share knowledge and support potential uptake of respite services by carers [39]. Low use of the My Aged Care website suggests the need to improve website usability, and/or provide greater support and training to promote self-efficacy and digital literacy in the broader population.

Previous research suggests that promoting the benefits of respite for both the carer and the person with dementia is important [12, 40] as well as service access features such as the availability of transport services, extended operating hours, and variety of activities available. Promotional messages should portray an inclusive and accepting attitude towards respite use. Respite service information should highlight the flexibility of services and the benefits of earlier and more respite frequent use [12]. Positive messaging could assist with addressing other barriers to respite use including beliefs regarding poor service quality, carer guilt or the possibility of negative outcomes. In a comprehensive strategy, other barriers such as poor quality and/or lack of availability and flexibility in service provision must also be addressed [13, 14]. The absence of a significant relationship between ‘wanting more respite’ and current respite information seeking behaviour suggests the need to address service deficits. However, this could also be related to the stress and strain experienced by carers with a high need for respite, whereby the sheer effort of actions involved in help-seeking becomes an additional burden they cannot manage [16].

In this study the relationship between the person with dementia’s behaviour with information seeking and higher unmet respite need highlights the significant physical and psychological strain on carers. Carers who reported receiving support to access services were less likely to report unmet needs. Dementia link workers have been found useful in supporting carers and people with dementia navigate and access services [41]. Psychoeducational interventions may also be useful to more effectively meet carer needs [42]. However, neither have been made available to Australian carers of people with dementia as part of new aged care reforms.

Finally, the results that carers who use more information sources have better knowledge of local services but more unmet need suggests that suitable respite services may not actually be available. In the context of new aged care reforms, this warrants further investigation and monitoring to determine whether reforms are delivering respite to those carers with the highest level of evaluated need.

### Limitations

As there are no national or state-based lists of carers of people with dementia living in the community, survey distribution used multiple distribution channels to reach the target group, with an original mail out to any carer who had used emergency respite over the past year. This meant surveys would have been received by recipients who may not have been in the target group as they were not caring for someone with dementia, or were previously in the target group but changed status in the previous year if the person they had been giving care to had moved into residential care or passed away, or the caregiver had moved address or passed away. Previous research has also found low response rates for postal surveys to older populations [43]. The estimated response rate of 18% appears reasonable with these considerations. Whilst it is acknowledged that this was a small regional study so may not be representative of carers in other parts of Australia, it provides critical insights into how the needs of a particularly vulnerable group of help-seeking carers are navigating a service system in transition. In doing so, it provides timely insights to inform system and policy adjustment in a timely fashion.

As this study explores the experience of carers navigating a service system in transition it may not be reflective of longer term outcomes assuming that carer knowledge and access improves as the community and health services adjust to the changes. This is particularly true of the new Carer Gateway (which commenced in Australia in December 2016) and was not evaluated as an information source in this study. Note however, the Carer Gateway has no funding to support the delivery of planned respite services and will only play a role in referring information seekers with a need in this area back to he My Aged Care website. However, it would be worthwhile reassessing carers’ knowledge, information seeking behaviours, and respite needs following the full implementation of the national aged care reforms.

## Conclusions

These findings indicate the new reforms in the Australian aged care sector have not adequately responded to the needs of carers of people with dementia for respite information and support. Whilst most carers reported seeking respite information, few had used the new government gateway or found them useful and instead were relying on interpersonal information sources. Respite seeking was associated with caring for someone with behavioural problems and was reinforced by the provision of personal support. In response to the high need for respite services, there is an urgent need to test the efficacy of additional interventions to work alongside current national programs to promote positive respite knowledge, attitudes and uptake among carers and people with dementia. Community-wide interventions, such as social marketing programs, can promote the benefits of respite and availability of respite services but this study demonstrated that personal support is preferred to navigate the system and source relevant respite information. The current findings highlight the importance of professional and personal channels, including the primary care sector and carer support groups, in increasing awareness of, and improving access to, respite services.

## Abbreviations

ACAT: Aged Care Assessment Team
CCRCs: Commonwealth Carer Respite Centres
CG: Care giver
GP: General practitioner
PWD: Person with dementia
